# Taxonomy of the *Cryptopygus* complex. II. Affinity of austral *Cryptopygus* s.s. and *Folsomia*, with the description of two new *Folsomia* species (Collembola, Isotomidae)

**DOI:** 10.3897/zookeys.658.11227

**Published:** 2017-02-28

**Authors:** Mikhail Potapov, Charlene Janion-Scheepers, Louis Deharveng

**Affiliations:** 1 Moscow State Pedagogical University, Kibalchicha str., 6, korp.3, Moscow, 129278, Russia; 2 School of Biological Sciences, Monash University, Victoria 3800, Australia; 3 Institut de Systématique, Evolution, Biodiversité, ISYEB – UMR 7205 – CNRS, MNHN, UPMC, EPHE, Museum national d’Histoire naturelle, Sorbonne Universités, 45 rue Buffon, CP50, F-75005 Paris, France

**Keywords:** Australia, New Zealand, taxonomy

## Abstract

*Folsomia minorae* **sp. n.** and *Folsomia australica* **sp. n.** are described from New Zealand and Australia, respectively. Their possible affinity to two different groups of *Cryptopygus* *sensu stricto* is discussed. Attention is paid to the variability of sensillary patterns of the genital segment in *Cryptopygus*: mainly, all s-chaetae are subequal, but in more advanced forms the dorsal triplet, lateral duplet or either of them become macrochaeta-like in length. *Cryptopygus ulrikeae* (= *Folsomia ulrikeae* Najt & Thibaud, 1987), **comb. n.** is given a new generic position.

## Introduction

The genus *Cryptopygus* Willem, 1902 *sensu stricto* has not received its modern generic diagnosis and most of its “austral” species need to be revised (Rusek 2002, Deharveng et al. 2005, Jordana et al. 2009, Potapov et al. 2013, Greenslade 2015). Several species related to *Cryptopygus antarcticus* Willem, 1902 (the type species of *Cryptopygus*) are common in the high latitudes of the Southern Hemisphere and form a clear taxonomic group. They clearly differ from other genera of the *Cryptopygus* complex that are found in more northern areas (tropical and further into the Northern Hemisphere). In this paper we temporarily define *Cryptopygus* *s.s.* as having 3 and 5 s-chaetae on Abd IV and V respectively, s-chaetae in mid-tergal position on body tergites, and having no differentiated foil-chaetae at the end of the abdomen. Whilst hemispheric distribution patterns of certain Collembola genera have been well documented (e.g. Holarctic for *Tetracanthella* Schött, 1891, see Deharveng 1987), this group of “austral” *Cryptopygus* (*Cryptopygus* *s.s.* below) can be considered a geographical equivalent of the mostly Holarctic genus *Folsomia* Willem, 1902. Although occurring in different hemispheres, both genera show an increasing diversity towards the poles, a similar set of life forms and play similar ecological roles in collembolan communities, which are more evident in polar zones. This geographical segregation is even further complicated by the movement of a few *Cryptopygus* *s.s.* and *Folsomia* to the opposite hemisphere. Until now, at least two “apparent” members of *Cryptopygus* *s.s.*, *Cryptopygus clavatus* (Schött, 1893) and *Cryptopygus roberti* (Fjellberg, 1991) are known from areas of the North Atlantic. Despite the strong similarity between these two forms and the main group of “austral” *Cryptopygus* the final generic position of *clavatus* and *roberti* remains unresolved. The state of a few native “austral” members of *Folsomia* is more obscure as important characters are lacking. Here we present two apparently native and new species from the Australian region. They are described and compared with “austral” *Cryptopygus* *s.s.*, the potential ancestors of “austral” *Folsomia*. These two species indicate that the latter genus possibly resulted from the convergent evolution of several species of the *Cryptopygus* *s.s.*

## Materials and methods

The notation system accp-as-al (Szeptycki 1972) was used to describe the set of s-chaetae. To establish the links between s-chaetae the approach by Potapov and Greenslade (2010) was applied to the genus *Folsomia*

### Abbreviations

**Abd. I–VI** abdominal segments I–VI

**accp** accessory p-row s-chaeta

**Ant. I–IV** antennal segments I–IV

**as** anterosubmedial s-chaeta

**a.s.l.** above sea level

**bms** basal micro s-chaeta on antennal segments

**e7** ‘guard’ of labial papilla E

**Leg I, II, III** first, second and third pairs of legs

**M** macrochaeta

**ms** micro s-chaeta(e) (=microsensillum(a) auct.)

**s** macro s-chaeta or s-chaetae (=macrosensillum(a) or sensillum(a) auct.)

***s.s.*** *sensu stricto*

**PAO** postantennal organ

**Th.II–III** thoracic segments II and III

**U3** inner edge of unguis

### Institutional acronyms

**MNZTPT** Museum of New Zealand Te Papa Tongarewa, Wellington

**SAMA** South Australian Museum, Adelaide

**MSPU** Moscow State Pedagogical University, Russia.

## Results

### Descriptions of new species

#### Folsomia minorae sp. n.

Taxon classificationAnimaliaEntomobryomorphaIsotomidae

http://zoobank.org/08169B5D-CB65-4F2C-9C35-93A8EE6F0C63

Figs 1–6 , 7–13

##### Type material.

Holotype: adult ♀. New Zealand, southern South Island, Central Otago, Pisa Range, 44°52'03"S, 169°9'33"E, 1700 m a.s.l., in soil and debris under *Dracophyllum muscoides* cushion, 18.ii.2014, coll. M. Minor (on slide). Paratypes. 10 paratypes, subadult ♀♀ and ♂♂of similar size with holotype, 7 of which from the same locality (and in close proximity), and 3 from Central Otago, The Remarkables Mts, 45°3'42"S, 168°48'40"E, 1829 m a.s.l., herbaceous snowbank, in soil, 19.ii.2014, coll. M. Minor (all on slides). Holotype and 4 paratypes kept in MNZTPT; 5 paratypes in MSPU.

##### Other material.

One ♀ identified in all details as *Folsomia minorae* sp. n. by A. Fjellberg (not seen by us): New Zealand, South Island (northern part), Avalanche Peak trail above Arthur Pass, 42°56'26"S, 171°33'29"E, forest litter, 23.i.2004, coll. A. Fjellberg.

##### Diagnosis.

*Folsomia* species with 5+5 ocelli; slender subapical organite of Ant.IV; clavate tibiotarsal hairs; outer teeth on claws; stout dens with few chaetae and a large mucro; and characteristic ‘3+2’ sensillary pattern of s-chaetae on Abd.V.

##### Description.

**Figures 1–6.**
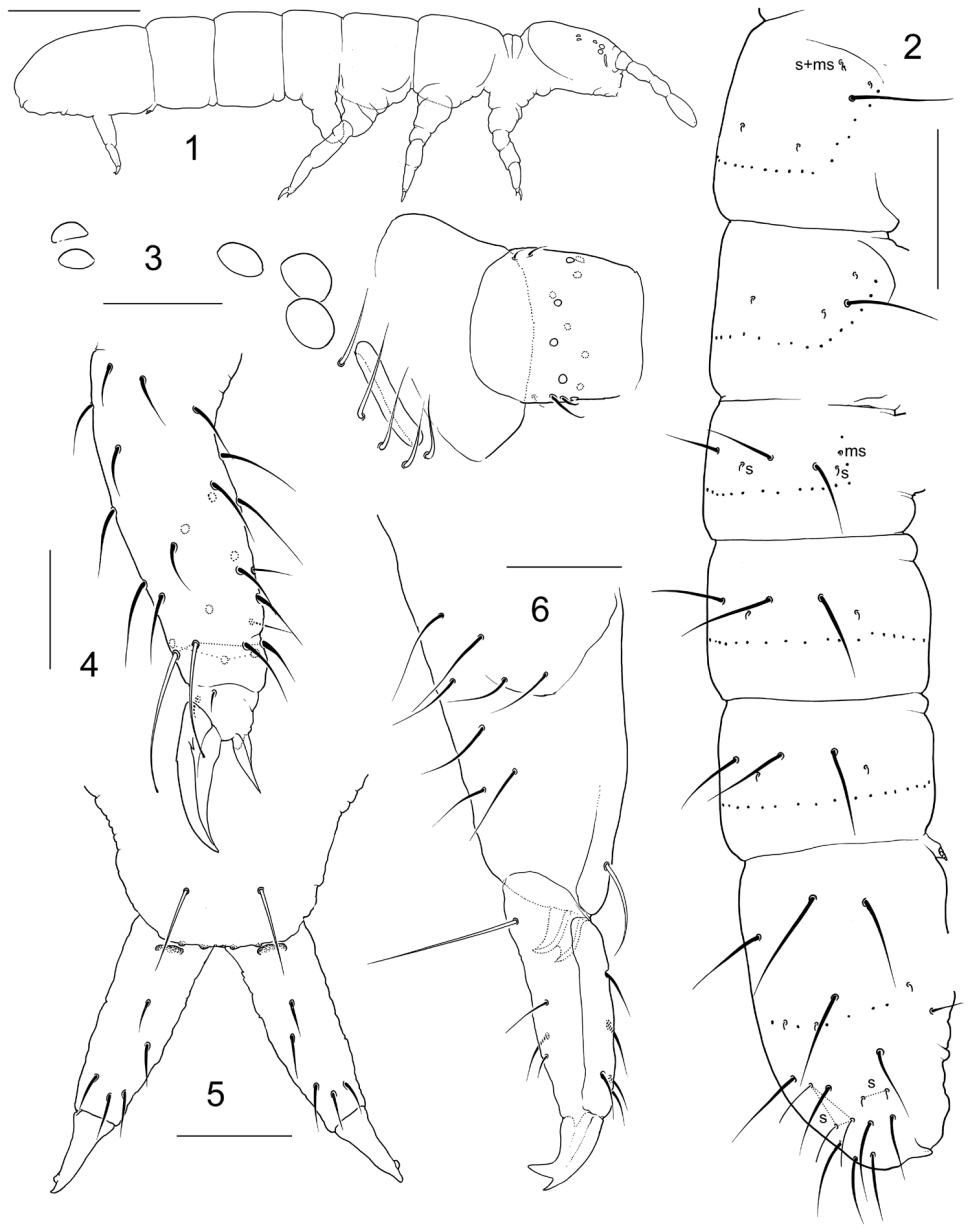
*Folsomia minorae* sp. n. **1** habitus **2** macrochaetae and s and ms-chaetae on body **3** anterior part of head **4** distal part of Leg III **5–6** furca, anterior (**5**) and lateral (**6**) view. Scale bar 0.3 mm in **1**; 0.15 mm in **2**, 0.03 mm in others.

**Figures 7–13.**
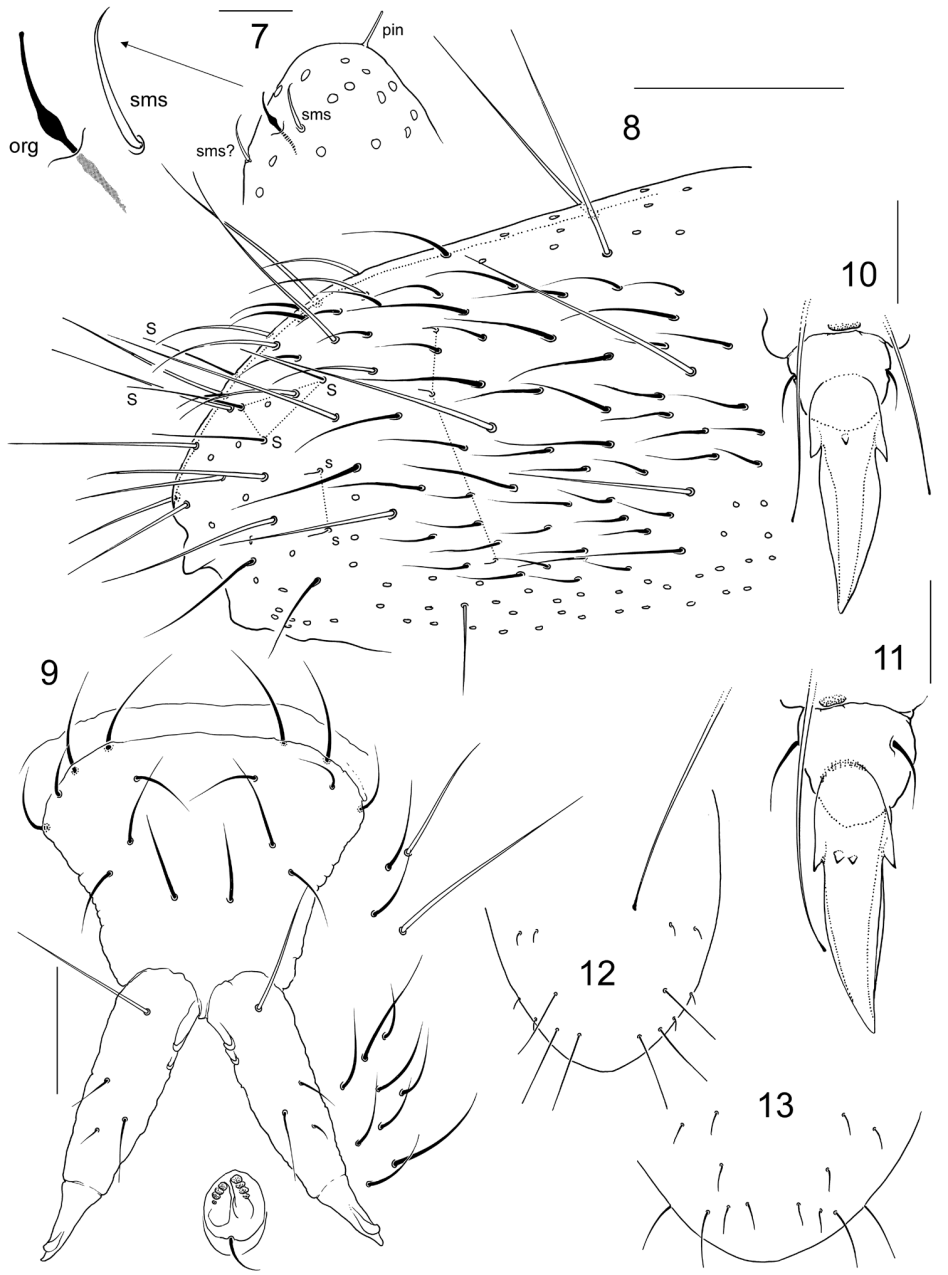
*Folsomia minorae* sp. n. (**7–12**) and *Folsomia australica* sp. n. (**13**) **7** apex of Ant.IV, lateral view **8** chaetotaxy of posterior part of Abd.IV, Abd.V and VI **9** furca, tenaculum, and furcal subcoxae, posterior view **10–11** apical part of Leg II (**10**) and III (**11**) **12–13** s-patterns chaetae of Abd.IV–V (lateral s of Abd.IV not shown). org—organite, sms—subapical ms, pin—pin-chaeta. Scal bar 0.1 mm in **8**, 0.03 mm in **9**, others, 0.01 mm.

Body size of the only adult female 1.75 mm. Dark blue, appendages paler. Body cylindrical (Fig. 1). Abd.IV, V and VI clearly fused dorsally, Abd.IV and III well separated. Cuticle reticulated, with roundish polygons, the largest of which almost as large as chaetae bases. Ocelli 5+5, arranged in two groups: three anterior and two posterior. PAO slender, not constricted, almost as long as width of Ant I (0.8–1.0) and 1.1–1.4 as long as inner unguis length (Fig. 3). Maxillary outer lobe with four sublobal hairs, maxillary palp bifurcate. Labral formula as 4/5,5,4. Labium with five papillae (A–E) and full set of ‘guards’, ‘guard’ e7 present, with three proximal, four basomedian, and five basolateral chaetae. Ventral side of head with 4+4 postlabial chaetae. Ant.I with two ventral s-chaetae (s) and three small basal ms-chaetae (bms), two dorsal and one ventral (Fig. 3), Ant.II with three bms and a latero-distal s, one of bms enlarged, Ant.III with one bms and with six distal s (including two lateral), without additional s-chaetae. S-chaetae on Ant.IV weakly differentiated. Apex of Ant.IV with two subapical ms (sms) both set at a distance from very long organite (org). Both sms of normal shape, organite with swelling in proximal part chili-shaped. The second subapical ms subequal to the first one, located more dorsally (Fig. 7). S-chaetae formula as common for the genus, 4,3/2,2,2,3,5 (s) and 1,0/1,0,0 (ms) (Fig. 2). Tergal s-chaetae much shorter than common chaetae and distinct. Medial s-chaetae on Th.II–Abd.III in mid-tergal position, on Abd.I–III between Mac1 and Mac2 (Fig. 2). Abd.V with five s-chaetae: three dorsal ones (al, accp1, accp2) long and slender, and two lateral short (‘3+2’ pattern) (Figs 2, 8, 12). Macrochaetae very long, stout and smooth, 1,1/3,3,3 in number, medial ones on Abd.V more than twice as long as dens (2.0–2.4) and 4.7–5.5 times longer than mucro. Foil chaetae at the tip of abdomen absent. Axial chaetotaxy of Th.II–Abd.III as 6–8,6–7/4,4,4. Thorax without ventral chaetae. Unguis stout, without inner teeth, with one (two on Leg III) outer and two large lateral teeth (Figs 4, 10, 11). The doubling of outer tooth on Unguis 3 well visible only in anterior position (Fig. 11). Empodial appendage about half as long as unguis (empodial appendage length: U3 = 0.46–0.53). Upper and lower subcoxae of Leg I, II, III with 1,1; 2–3,6; 4–5,6–9 chaetae. Tibiotarsi without additional chaetae on Leg I and II (21 chaetae), and with several additional chaetae on Leg III (more than 26 at whole). Tibiotarsal tenent chaetae clavate, long (1.3–1.6 longer than inner edge of U3), in number 1, 2, 2 on Leg I, II, III. VT with 4+4 laterodistal and 6 posterior chaetae, anteriorly without chaetae. Laterodistal chaetae arranged almost in a line, posterior chaetae in two rows, proximal (2) and distal (4). Tenaculum with 4+4 teeth and one chaeta. Basal tooth smaller than others (Fig. 9). Anterior furcal subcoxae with 8–9 chaetae, posterior one with four chaetae. Anterior side of manubrium with a pair of chaetae (Fig. 5). Posterior side of manubrium with 4+4 laterobasal and 4+4 on main part, without apical and lateral chaetae. To describe chaetae on main part the notation system of Fjellberg (2007) can be somewhat applied: chaetae M1, M2, pr and ml1 present (Fig. 9). Dens stout, with five anterior chaetae arranged as 1,1,3, the second single chaeta positioned more medially than the first (Figs 5, 6). Posterior side of dens almost smooth, with four chaetae of which one strong basal and three in central part (two of normal size and one small) (Fig. 9). Very large, chitinized, bidentate (Figs 6, 9). Ratio of manubrium : dens : mucro = 3.6–4.2 : 2.0–2.4 : 1.

##### Etymology.

The name is given after Maria Minor, who kindly provided some of the material on the new species.

##### Discussion.

To date eight species of *Folsomia* are known from New Zealand (Greenslade 1994; 2012). In addition, three species, *Folsomia parasitica* Salmon, 1942, *Folsomia novaezealandiae* Salmon, 1943, and *Folsomia lunata* Salmon, 1943, were removed from the list as synonyms or were moved to the genus *Cryptopygus* (Bellinger et al. 2016). Among the valid species, five are blind, while others show different number of ocelli (8, 2 and 1, vs. 5 in *Folsomia minorae* sp. n.). Very little morphological data are available for endemic New Zealand *Folsomia* species (*Folsomia miradentata* Salmon, 1943, *Folsomia pusilla* Salmon, 1944, *Folsomia salmoni* Stach, 1947, and *Folsomia sedecimoculata* Salmon, 1943). Particularly, figures of the furca are known only for *Folsomia sedecimoculata* and *Folsomia pusilla*. Both species show a more common structure of the dens (typical of the genus), which is slender and continuously narrowed, unlike in *Folsomia minorae* sp. n. Clavate tibiotarsal hairs were not figured or mentioned in descriptions of New Zealand forms (present in the new species) . A comparison between *Folsomia minorae* sp. n. and *Cryptopygus* *s.s.* is given below.

Differentiating characters of the new species are: five ocelli, unique subapical organite of Ant.IV, clavate tibiotarsal hairs, presence of outer teeth on claws, stout dens with few chaetae, and a very large mucro. Well differentiated ‘3+2’ sensillary pattern of s-chaetae on Abd.V is also characteristic (see below). Three long and slender dorsal s-chaetae of Abd.V are found in species of several groups of *Folsomia* of the Holarctic (i.e. *Folsomia penicula* Bagnall, 1939, *Folsomia quadrioculata* (Tullberg, 1871) and *Folsomia sensibilis* Kseneman, 1936), which belong to either ‘3+2’ or ‘3+1+1’ patterns.

##### Distribution and ecology.

*Folsomia minorae* sp. n. is known from three localities in South Island, New Zealand. It is probably a species restricted to mountainous areas.

#### Folsomia australica sp. n.

Taxon classificationAnimaliaEntomobryomorphaIsotomidae

http://zoobank.org/C9C7BCD0-5200-4701-A90A-7F2763C9A550

Figs 14–21

##### Type material.

Holotype: adult ♀. Australia, Chiltern National Park, Victoria, heathy dry forest, mostly native, 36°7'53"S, 146°36'20"E, 12.iv.2015, M. Lythe leg. Four paratypes, one adult ♀, one adult ♂and two sub-adult ♀♀ of the same size as adults. Holotype and two paratypes in SAMA, two paratypes in MSPU.

##### Other material.

10 specimens in ethanol (SAMA). Australia, Victoria, Mt Pilot National Park, 36°18'45"S, 146°33'16"E. 20.vii.2015, M. Lythe leg.

##### Diagnosis.

*Folsomia* species with 1+1 ocelli; chaetotaxy of dens 12/6; tridentate mucro; 2 lateral s-chaetae on Abd.V clearly longer than 3 dorsal ones; 2+2 chaetae on anterior side of manubrium.

##### Description.

**Figures 14–21.**
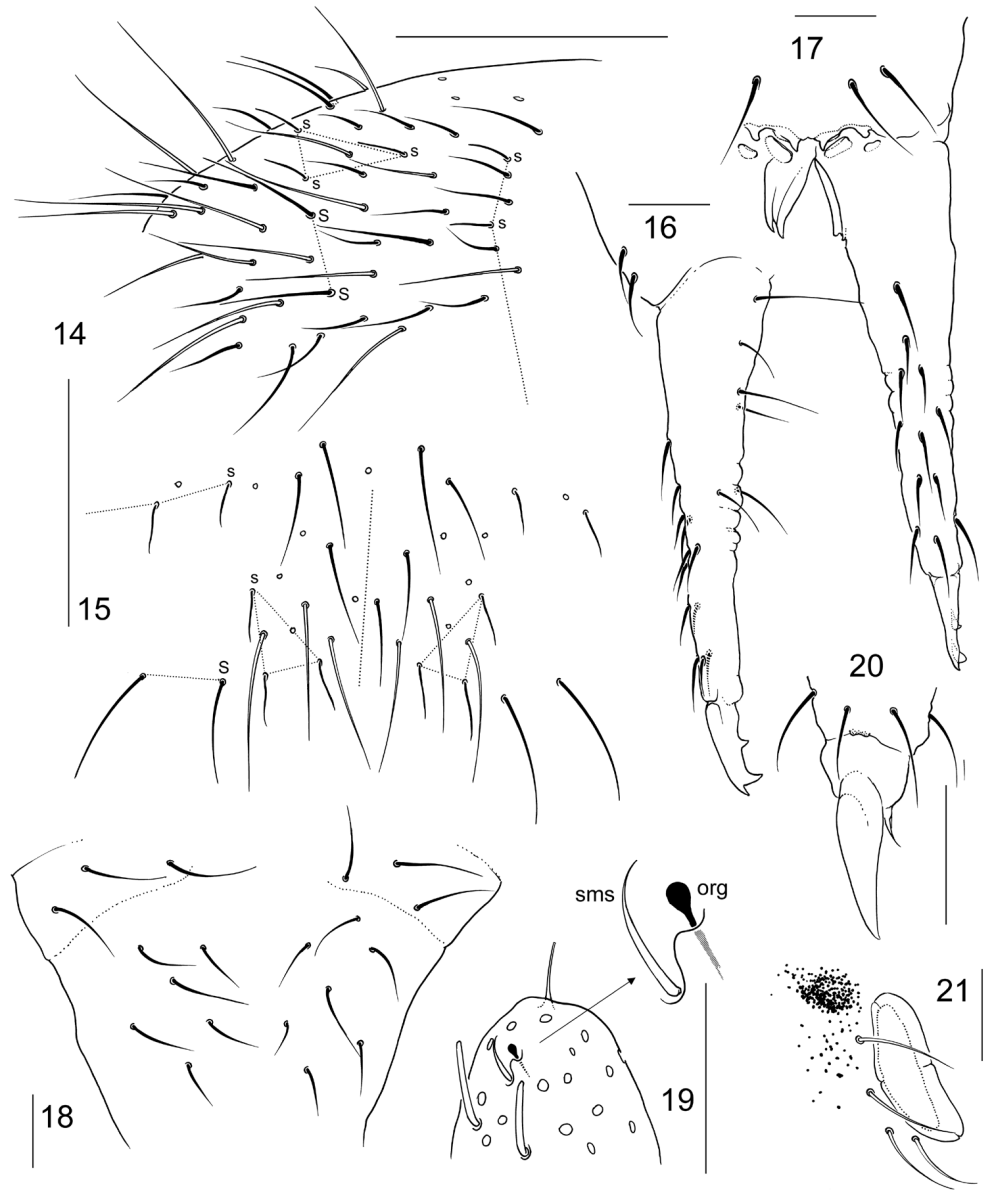
*Folsomia australica* sp. n. **14–15** chaetotaxy of posterior part of abdomen, lateral (**14**) and dorsal (**15**) views **16–17** furca, lateral (**16**) and anterior (**17**) view **18** manubrium, posterior view **19** apex of Ant.IV, dorsal view, left antenna **20** distal part of Leg III **21** ocellus and PAO. org—organite, sms—subapical ms. Scale bar 0.05 mm in **14, 15**, others, 0.01 mm.

Body size from 0.60 (adult male) to 0.75 mm (one of sub-adult females). White, with one black ocellus on each side of the head (Fig. 21). Body of normal shape for the genus. Abd IV, V and VI clearly fused dorsally, Abd.IV and III well separated. Cuticle “smooth”, with fine orthogonal granulation, granules much smaller than chaetae bases. Ocelli 1+1, well-marked only by pigmentation, cuticular cornea weak. PAO wide, constricted, smaller (ca. 0.8) than width of Ant I, about 1.5 as long as inner unguis length (Fig. 21). Maxillary outer lobe with four sublobal hairs, maxillary palp bifurcate. Labral formula as 4/5,5,4. Labium with five papillae (A–E), ‘guard’ e7 present (whole number of ‘guards’ hard to estimate), with three proximal, four basomedian, and five basolateral chaetae. Ventral side of head with 4+4 postlabial chaetae. Ant.I with three ventral s-chaetae (s) and two small basal ms-chaetae (bms), dorsal and ventral, Ant.II with three bms and one latero-distal s. Ant.III with one bms and with five distal s (including one lateral), without additional s-chaetae. Ant.IV with several tubular s-chaetae. Subapical organite large and roundish, set together with subapical ms, as common for family (Fig. 19). S-chaetae formula 4,3/2,2,2,3,5 (s) and 1,0/0,0,0 (ms). Tergal s-chaetae shorter than common chaetae. Medial s-chaetae on Th.II–Abd.III in mid-tergal position, on Abd.I–III between Mac1 and Mac2. Abd.V with five s-chaetae with three dorsal ones (al, accp1, accp2), almost as long as common chaetae, and two lateral long, macrochaetae-like (‘3+2’ pattern) (Figs 14–15). Two lateral s-chaetae often slightly thickened on proximal 2/3 that makes them more distinct. Macrochaetae smooth, 1,1/3,3,3 in number, medial ones on Abd.V shorter than dens (0.6–0.8) and 2.6–3.0 times longer than mucro. Foil chaetae at the tip of abdomen absent. Axial chaetotaxy of Th.II–Abd.III as 9–10,6–8/4–5,4–5,4. Th.III with 1+1 ventral chaetae. Unguis without teeth (Fig. 20). Empodial appendage about 0.6 as long as U3. Upper and lower subcoxae of Leg I, II, III with 1,1; 3,6; 5–6,6–7 chaetae. Tibiotarsi without additional chaetae on Leg I and II (21 chaetae), and with several additional chaetae on Leg III. Tibiotarsal tenent chaetae pointed, shorter than U3 (0.8–1.0). VT with 3+3 laterodistal and five posterior chaetae, of which four in transversal row, anteriorly without chaetae. Tenaculum with 4+4 teeth and a chaeta. Anterior furcal subcoxae with 8–12, posterior one with five chaetae. Anterior side of manubrium with two pair of chaetae, 2+2 (rarely 1+2) (Figs 16, 17). Posterior side of manubrium with 3+3 laterobasal, 6-7+6-7 on main part, without apical and lateral chaetae (Fig. 18) (shown in the only variant seen). Dens slender, with 12 anterior chaetae arranged as 1,1,2,3,2,3 (Figs 16, 17). Posterior side of dens with few distinct crenulations at the middle, four chaetae on proximal half and two medially. Mucro tridentate. Ratio of manubrium : dens : mucro = 2.9–3.4 : 3.6–4.2 : 1.

##### Etymology.

The name is given after the geographical distribution of the new species.

##### Discussion.

*Folsomia australica* sp. n. resembles the only other native Australian species of the genus, i.e. *Folsomia loftyensis* (Womersley, 1934) (after the redescription of Potapov and Greenslade 2010) by chaetotaxy of dens 12/6, tridentate mucro, 1+1 ocelli, ms-formula of body 10/000, differentiation of s-chaetae on Abd.V, and other characters. It differs in having 2+2 chaetae (vs. 4–5+4–5 in *Folsomia loftyensis*) on the anterior side of manubrium. Juvenile specimens of the two species are probably hard to distinguish. The new species was recorded by Potapov and Greenslade (2010) as “Folsomia sp. aff. loftyensis”. *Folsomia australica* sp. n. and *Folsomia minorae* sp. n. are dissimilar indicating that the “austral “ members of the genus *Folsomia* can also be heterogeneous, as in the Northern Hemisphere.

Morphological features of the furca of *Folsomia australica* and *Folsomia loftyensis*, especially the tridentate mucro, are shared with several species of *Cryptopygus: Cryptopygus tricuspis* Enderlein, 1909 (sub-Antarctic), *Cryptopygus insignis* Massoud and Rapoport, 1968 (South America), *Cryptopygus patagonicus* Izarra, 1972 (South America), and three unnamed species from South Africa (*Cryptopygus* sp. 5, *Cryptopygus* sp. 6, and *Cryptopygus* sp. 7, see below). These species probably represent another group of *Cryptopygus* *s.s.*, dissimilar to *Cryptopygus antarcticus*, which could be ancestral to the “Australian” species of Folsomia.

##### Distribution.

*Folsomia australica* sp. n. is known from two localities in south-eastern part of Australia (Victoria and New South Wales).

## General discussion

**Figures 22–25.**
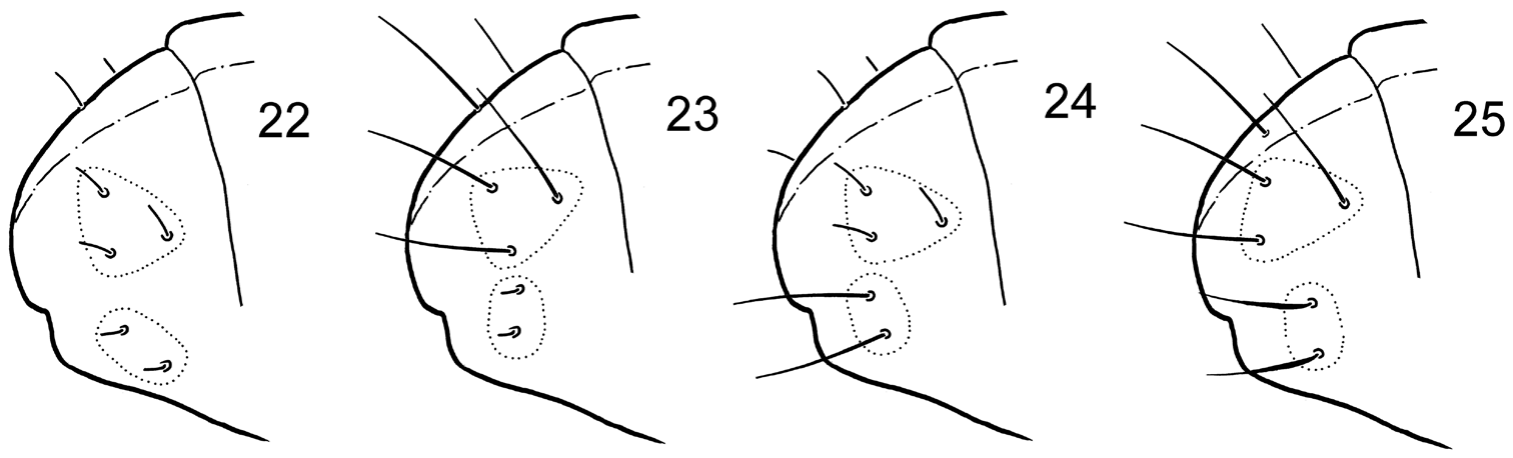
“3+2” s-patterns of austral *Cryptopygus*. **22** *Cryptopygus antarcticus* **23–25** *Cryptopygus*: sp. 5 (**23**) sp. 6 (**24**) sp. 7 (**25**) (all three species from South Africa).

**Figures 26–29.**
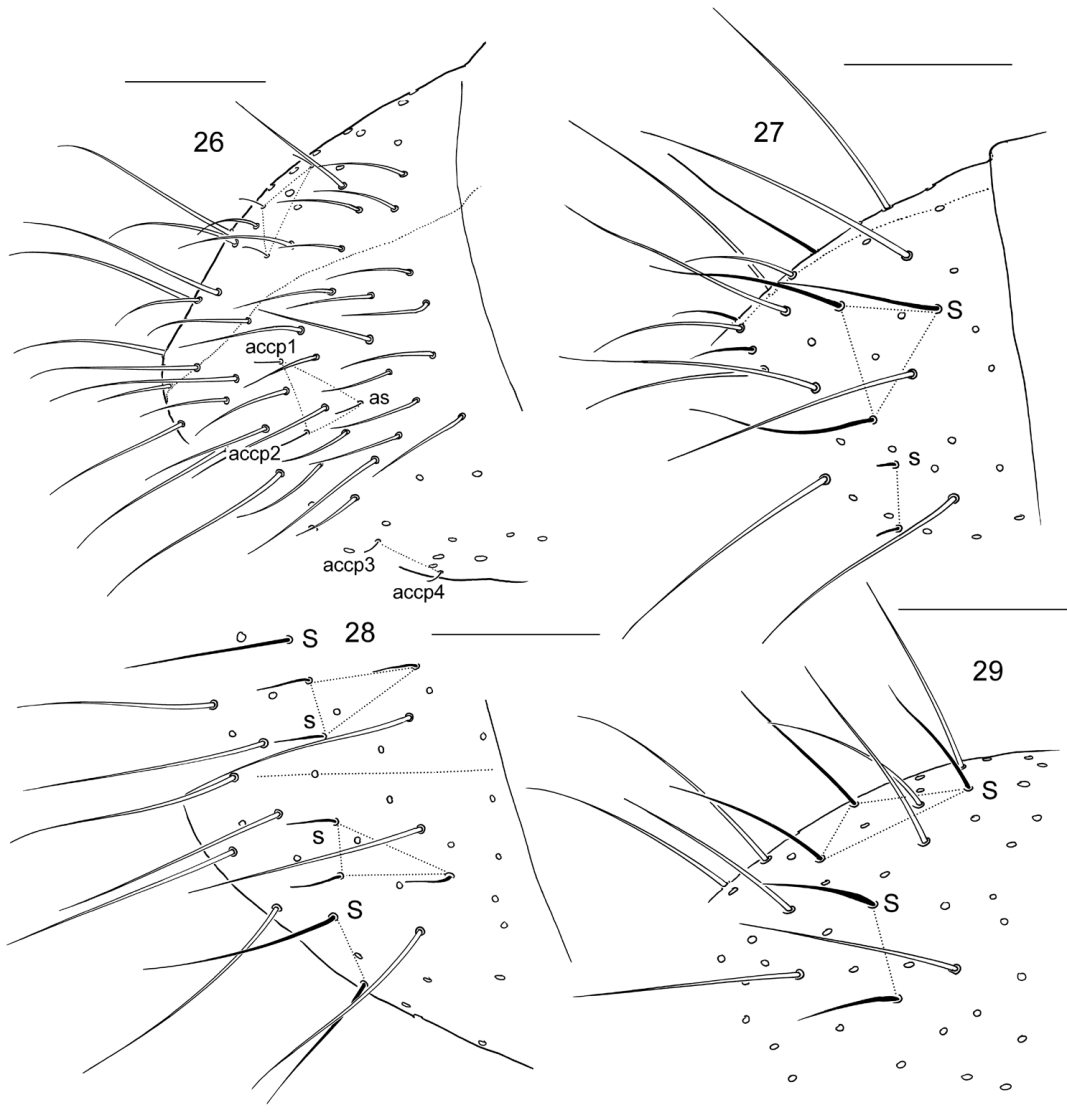
S-chaetae on Abd.V in austral *Cryptopygus*. **26** *Cryptopygus antarcticus* **27–29** *Cryptopygus*: sp.5 (**27**) sp.6 (**28**) sp.7 (**29**) (all three species from South Africa). Scale bar 0.05 mm.

The fifth abdominal segment of all species of the genus *Cryptopygus*, as in *Folsomia*, has five s-chaetae on each side: accp1, accp2, accp3, accp4 and as. The s-pattern of *Cryptopygus antarcticus* and several other species is probably the most primitive as it consists of regularly scattered sub-equal short and thin s-chaetae (Fig. 26). Weak differentiation is observed: s-chaetae of dorsal triplet (as+accp1+accp2) slightly longer and sometimes thinner than s-chaetae of lateral duplet (accp1+accp2) (Figs 22, 26). Such arrangement and differentiation of s-chaetae can be called as a weak ‘3+2’ pattern (for terminology, see Potapov and Greenslade 2010). Most species of *Cryptopygus* *s.s.* show this pattern, sometimes the difference between the length of “triplet” and “duplet” s-chaetae is hardly evident. Subsequent evolution is expressed by stronger differentiation: s-chaetae of dorsal triplet, lateral duplet or either of them become macrochaeta-like (Figs 23–25). Only one species for each of these three apomorphic conditions was found in the material studied here, all only currently known from South Africa. The representation of s-patterns in *Cryptopygus* *s.s.* for Abd.V is as follows:

1. Weakly differentiated “3+2” pattern (Figs 22, 26). S-chaetae shorter than common chaetae. In a few short-haired species, (*Cryptopygus badasa* Greenslade, 1995, *Cryptopygus binoculatus* Deharveng, 1981, *Cryptopygus lawrencei* Deharveng, 1981, *Cryptopygus* sp.4) s-chaetae are almost as long as common chaetae.

Representatives:

– *Cryptopygus antarcticus antarcticus* Willem, 1902. Material: several locations in Antarctic Peninsula (King George Isl., Deception Isl., Devil Isl.) leg. D. Russell.

– *Cryptopygus antarcticus maximus* Deharveng, 1981. S-chaetae as in nominate subspecies. Material: Kerguelen Isl. leg. J. Travé.

– *Cryptopygus antarcticus reagens* (Enderlein, 1909). S-chaetae of dorsal triplet almost twice longer than s-chaetae of duplet. Material: Crozet Isl. (sub-Antarctic), leg. J. Travé.

– *Cryptopygus antarcticus travei* Deharveng, 1981. S-chaetae of dorsal triplet almost as long as those of duplet. Material: Marion Isl. (sub-Antarctic), leg. J. Travé.

– *Cryptopygus araucanus* Massoud & Rapoport, 1968. S-chaetae of dorsal triplet slightly longer than s-chaetae of duplet. Material: syntypes, several locations in Argentina (Futalaufquen, Lago Curruhé, Lago Menendez). Collections of the Museum national d’Histoire naturelle (Paris, France).

– *Cryptopygus badasa* Greenslade, 1995. All s-chaetae very short, “triplet” s-chaetae slightly longer. Material: Antarctic Peninsula (Devils Isl.), leg. D. Russell; South Georgia (sub-Antarctic), leg. V. Bulavintsev.

– *Cryptopygus binoculatus* Deharveng, 1981. S-chaetae subequal. Material: holotype, Crozet Isl. (sub-Antarctic).

– *Cryptopygus insignis* Massoud & Rapoport, 1968. S-chaetae subequal. Material: syntypes, Lago Menendez (Argentina). Collections of the Museum national d’Histoire naturelle (Paris, France).

– *Cryptopygus hirsutus* (Denis, 1931). S-chaetae subequal. Material: possible syntypes, Costa Rica. Collections of the Museum national d’Histoire naturelle (Paris, France).

– *Cryptopygus lawrencei* Deharveng, 1981. “Triplet” s-chaetae slightly longer. Material: Kerguelen Isl. (sub-Antarctic), leg. J. Travé.

– *Cryptopygus pilosus* (Womersley, 1934). S-chaetae as in *Cryptopygus antarcticus*. Material: South Australia, Lofty Ranges, leg. P. Greenslade.

– *Cryptopygus tricuspis* Enderlein, 1909. S-chaetae as in *Cryptopygus antarcticus*. Material: Kerguelen Isl. (sub-Antarctic), leg. J. Travé.

– *Cryptopygus ulrikeae* (Najt & Thibaud, 1987), **comb. n.** S-chaetae sub-equal. Separation of Abd.IV and V as in other species of the genus *Cryptopygus*. Primarily, it was described as *Folsomia ulrikeae* (Najt and Thibaud, 1987). Material: holotype, Ecuador. Collections of the Museum national d’Histoire naturelle (Paris, France).

– *Cryptopygus* sp. 1 (complex ‘antarcticus’). S-chaetae as in *Cryptopygus antarcticus*. Characters common with the nominotypic subspecies of *Cryptopygus antarcticus* but body more slender. Material: New Zealand (South Island), leg. M. Minor.

– *Cryptopygus* sp. 2 (complex ‘antarcticus’). S-chaetae as in *Cryptopygus antarcticus*. With the characters of *Cryptopygus antarcticus* but ms formula of body tergites as 10/000. Material: New Zealand (North Island).

– *Cryptopygus* sp. 3. S-chaetae as in *Cryptopygus antarcticus*. With 6+6 ocelli. Manubrium without anterior chaetae Dens with one anterior chaeta, mucro bidentate. Material: South Africa (Jonkershoek), leg. Cryptopygus Janion-Scheepers.

– *Cryptopygus* sp. 4. S-chaetae as in *Cryptopygus antarcticus*. With 8+8 ocelli. Dens rather long, with nine anterior and 5 posterior chaetae, mucro bidentate. Common chaetae and macrochaetae on body short. Material: South Africa, (Sutherland), leg. Cryptopygus Janion-Scheepers.

2. “3+2” pattern with development of dorsal triplet (Figs 23, 27). Three dorsal s-chaetae (as, accp1, accp2) almost as long as macrochaetae, two lateral (accp3, accp4) short.

– *Cryptopygus* sp. 5. Dens with 9-10 anterior and 6 posterior chaeta, mucro tridentate. Material: South Africa (Table Mountain), leg. L. Deharveng and A. Bedos.

3. “3+2” pattern with development of lateral duplet (Figs 24, 28). Three dorsal s-chaetae (as, accp1, accp2) short, two lateral (accp3, accp4) long.

– *Cryptopygus* sp.6. With 4+4 or 5+5 ocelli. PAO with strong inner denticles. Dens with ten anterior chaetae, mucro tridentate. Material: South Africa (Little Switzerland), leg. E. Krzemińska.

4. “3+2” pattern with development of all s-chaetae (Figs 25, 29). Three dorsal s-chaetae (as, accp1, accp2) thin, two lateral (accp3, accp4) also long, somewhat shorter than dorsal, somewhat flame-shaped.

– *Cryptopygus* sp.7. With 4+4 ocelli. Dens with 11-12 anterior and five posterior chaeta, mucro tridentate. Material: South Africa (Sutherland), leg. Cryptopygus Janion-Scheepers.

S-chaetae patterns of *Cryptopygus* are probably more diverse than shown above: *Cryptopygus yosii* Izarra, 1965 (Argentina, after our study of a syntype) shows “3+1+1” pattern in which accp3 is thick and tubular and accp4 is short and moved to the latero-ventral position. More material on less primitive species needs to be studied to complete the generic overview. Nevertheless, s-patterns of Abd.V in *Cryptopygus* seem to be less divergent than in the larger genus *Folsomia* (Potapov and Greenslade 2010), while “austral” variant 4 (see above) has not been discovered in the latter genus.

The dorsal fusion or separation of genital (Abd.V) and pre-genital segment (Abd.IV) is traditionally considered to be of great taxonomic value in the classification of the subfamily Anurophorinae *s.l.*, and the genus *Folsomia* is defined by the apomorphic condition of this character (fusion). Based on the available literature and our own observations, the s-chaetotaxy of “austral” *Cryptopygus* *s.s.* shows principally the same characteristics as in *Folsomia*, particularly 4,3/2,2,2,3,5 set and arrangement of s-chaetae on Abd.V. The more adaptive characters (furca, ocelli, etc.) vary considerably within both genera. Therefore Abd.IV–V fusion seems to be the only apomorphic character that separates *Folsomia* from *Cryptopygus* and the former genus can be easily derived from the latter. This key character can potentially show a high level of homoplasy and *Folsomia* is probably a polyphyletic or paraphyletic group. In the Northern Hemisphere, the high diversity of *Folsomia* makes it difficult to find an appropriate ancestor or even ancestors among known taxa. In contrast, at least three “austral” native *Folsomia* mentioned above show much in common with certain species of *Cryptopygus* *s.s.* Thus, all the main characters of *Folsomia minorae* sp. n. (ocelli, clavate tibiotarsal hairs, outer teeth on claws, dens and mucro) indicate its close relationship to a group of species similar to *Cryptopygus antarcticus* (Wise 1967; Massoud and Rapoport 1968, Deharveng 1981), while the characters of *Folsomia australica* sp. n. are shared with several *Cryptopygus* species with a slender dens and tridentate mucro (see the remarks to the species). S-chaetae patterns of Abd. V in *Folsomia minorae* is the same as in *Cryptopygus* sp. 5 from South Africa (Figs 12, 23), to which this new species is, however, less similar than to the “antarcticus” group. S-pattern of *Folsomia australica* and *Folsomia loftyensis* is identical to *Cryptopygus* sp. 6 from South Africa (Figs 14, 24).

The generic position of both lineages, *Folsomia minorae* and *Folsomia australica-loftyensis*, can be modified in the future, depending on the increase of knowledge on the generic groups “*Cryptopygus*” and “*Folsomia*”. The genus *Folsomia* is also very diverse in the Holarctic and consists of several species groups of which several differ in characters of great taxonomical value and may justify the status of new separate genera.

## Supplementary Material

XML Treatment for Folsomia minorae

XML Treatment for Folsomia australica
